# A Sex-Specific Comparative Analysis of Oxidative Stress Biomarkers Predicting the Risk of Cardiovascular Events and All-Cause Mortality in the General Population: A Prospective Cohort Study

**DOI:** 10.3390/antiox12030690

**Published:** 2023-03-10

**Authors:** Martin F. Bourgonje, Amaal E. Abdulle, Lyanne M. Kieneker, Sacha la Bastide-van Gemert, Stephan J. L. Bakker, Ron T. Gansevoort, Sanne J. Gordijn, Harry van Goor, Arno R. Bourgonje

**Affiliations:** 1 Department of Pathology and Medical Biology, University Medical Center Groningen, University of Groningen, 9713 GZ Groningen, The Netherlands; 2 Department of Internal Medicine, Division of Vascular Medicine, University Medical Center Groningen, Medicine, University of Groningen, 9713 GZ Groningen, The Netherlands; 3 Department of Internal Medicine, Division of Nephrology, University Medical Center Groningen, University of Groningen, 9713 GZ Groningen, The Netherlands; 4 Department of Epidemiology, University Medical Center Groningen, University of Groningen, 9713 GZ Groningen, The Netherlands; 5 Department of Obstetrics and Gynecology, University Medical Center Groningen, University of Groningen, 9713 GZ Groningen, The Netherlands; 6 Department of Gastroenterology and Hepatology, University Medical Center Groningen, University of Groningen, 9713 GZ Groningen, The Netherlands

**Keywords:** oxidative stress, biomarkers, cardiovascular disease, mortality, population study

## Abstract

Oxidative stress plays a pivotal role in cardiovascular (CV) disease, but current biomarkers used to predict CV events are still insufficient. In this study, we comparatively assessed the utility of redox-related biomarkers in predicting the risk of CV events and all-cause mortality in male and female subjects from the general population. Subjects (*n* = 5955) of the Prevention of REnal and Vascular ENd-stage Disease (PREVEND) population-based cohort study were included. Blood homocysteine, gamma-GT, HDL cholesterol, bilirubin and protein-adjusted free thiol (R-SH, sulfhydryl groups) levels were quantified at baseline and were prospectively analyzed in association with the risk of CV events and all-cause mortality. After adjustment for potentially confounding factors, protein-adjusted R-SH and homocysteine levels were significantly associated with the risk of CV events in men (HR 0.63 [0.40–0.99], *p* = 0.045 and HR 1.58 [1.20–2.08], *p* = 0.001, respectively). Protein-adjusted R-SH and HDL cholesterol levels were significantly associated with the risk of all-cause mortality in men (HR 0.52 [0.32–0.85], *p* = 0.009 and HR 0.90 [0.85–0.94], *p* < 0.001, respectively), while the same was observed for bilirubin and homocysteine levels in women (HR 0.68 [0.48–0.98], *p* = 0.040 and HR 2.30 [1.14–3.76], *p* < 0.001, respectively). Lower levels of protein-adjusted R-SH were robustly associated with an increased risk of CV events and all-cause mortality in men. Our results highlight the value of R-SH levels in cardiovascular risk assessment and their potential significance as being amenable to therapeutic intervention, while reaffirming the importance of other oxidative stress-related biomarkers, such as homocysteine, HDL cholesterol and bilirubin.

## 1. Introduction

Oxidative stress plays an important role in the pathogenesis of many conditions, such as aging, cardiovascular disease, diabetes, and metabolic-associated fatty liver disease [1]. Understanding the role of oxidative stress in these diseases could be paramount to the detection, treatment and prevention of disease. It is defined as an imbalance between oxidants and antioxidants in favor of the oxidants, leading to a disruption of redox signaling and control, and/or molecular damage [2]. Reactive oxygen species (ROS) play a pivotal role in the response to hypoxia, inflammation, and various physiological systems, such as the regulation of immunity, differentiation, longevity, and autophagy of cells [3]. Oxidative stress is a key effector mechanism in the pathophysiology of numerous inflammatory and hypoxic conditions, and is closely associated with systemic inflammation, which can culminate into oxidative damage across all levels of biological organization [4]. It has been reported to play a role in several risk factors of cardiovascular disease, such as hypertension, obesity, diabetes, metabolic syndrome, dyslipidemia, and peripheral arterial disease [5].

Various plasma and serum markers of oxidative stress have been studied in health and disease, in order to determine their relation to disease and disease severity and to examine their predictive value. Among these are serum free thiol groups (sulfhydryl groups, R-SH), which are considered to be representative biomarkers of systemic oxidative stress [6]. Free thiols play a pivotal role in extracellular antioxidant systems, and possess potent antioxidant activity [7]. Reduced levels of serum free thiols arise from rapid oxidation by high amounts of ROS, and can be indicative of an unfavorable redox status, whereas higher concentrations of serum free thiols are indicative of a more favorable redox status. Systemic redox status has been shown to be associated with disease severity, and is a predictor of clinical outcomes in multiple conditions [8,9,10,11]. Homocysteine, a homologue of the amino acid cysteine, is also considered to be a representative systemic biomarker for oxidative stress [12]. High levels of homocysteine are especially considered to comprise an important risk factor for cardiovascular disease [13]. High-density-lipoprotein (HDL) cholesterol has been shown to strongly and inversely correlate with cardiovascular disease, and is known for its anti-inflammatory, anti-thrombotic, and anti-oxidative properties [14,15]. Bilirubin, a product of heme catabolism, is widely accepted as a biochemical indicator for the diagnosis of blood system diseases, as well as liver and bile diseases. In addition to this, bilirubin is considered to be a powerful antioxidant, and forms an important component of total antioxidant capacity in addition to free thiol groups [16]. While bilirubin is also known to exert cytotoxic effects in higher concentrations, concentrations in the physiological range only appear to exert antioxidant effects [17]. Gamma-glutamyl transferase (γ-GT) is an enzyme that catalyzes the breakdown of (among other molecules) glutathione, an important antioxidant molecule. Elevated levels of γ-GT have been linked to an increased risk of various diseases, such as diabetes and cardiovascular disease [18,19].

All these systemic biomarkers of oxidative stress have not only been shown to be cardinal in various disease conditions, but have also been shown to have value as predictors of health outcomes in the general population [20]. Currently it is unknown as to which of these biomarkers of oxidative stress is associated most strongly with the risk of CV events and all-cause mortality. Additionally, potential sex-specific associations between oxidative stress biomarkers and the risk of adverse health outcomes have not been properly investigated yet. Cardiovascular disease and all-cause mortality can vary between men and women in incidence, prevalence, etiology and morbidity, and to understand the underlying pathophysiological mechanisms, it is important to study potential sex-specific associations, as well as any potential combined associations [21,22,23].

In the present study, we aimed to determine as to which of the aforementioned systemic biomarkers, alone or in combination, associates best with the risk of cardiovascular events and all-cause mortality, in both male and female individuals from the general population. Therefore, we aimed to comparatively assess the utility of these five potential biomarkers in predicting the sex-specific risk of cardiovascular events and all-cause mortality.

## 2. Materials and Methods

### 2.1. Study Population

The PREVEND (Prevention of REnal and Vascular ENd-stage Disease) study is a large-scale, prospective cohort study based in the city of Groningen, the Netherlands [24]. It was initiated in 1997 to investigate the relationship between albuminuria and the occurrence of renal and cardiovascular diseases. It collected data on a large number of variables from individuals living in Groningen who were between the ages of 28 and 75 years. A total of 85,421 people filled in a questionnaire and collected a urine sample, and a total of 40,856 subjects (47.8%) completed both. Of these, participants with urinary albumin concentrations (UAC) > 10 mg/L (*n* = 7786) and a randomly selected control group with UAC < 10 mg/L (*n* = 3395) were invited to participate in subsequent study investigations at the research clinic of the University Medical Center Groningen (UMCG). The questionnaire featured data on demographic variables, history of cardiovascular disease, pregnancy history, and medication usage. This second screening program was completed by 8592 participants (*n* = 6000 with UAC > 10 mg/L and *n* = 2592 with UAC < 10 mg/L), which together formed the full PREVEND study cohort. Participants that were excluded were subjects who were pregnant, had Type 1 diabetes, or who had insulin-treated Type 2 diabetes. Another visit was initiated between 2001 and 2003 to collect a second set of serum samples from 6136 of these participants. For the present study, the data from this second visit were used as a baseline. Participants with cardiovascular (CV) events between the first and second visit (*n* = 181) were excluded from the study, since re-events were not registered for these individuals. This resulted in a total of 5955 participants included in this study. The study was reviewed by the Institutional Review Board (IRB) of the UMCG (MEC 96/01/022). All eligible individuals gave written informed consent for their participation, and the study was performed according to the Declaration of Helsinki (2013) principles.

### 2.2. Data Collection

All patients filled out a questionnaire pertaining to information regarding their demographics, lifestyle habits (e.g., smoking, alcohol consumption), health status (e.g., history of cardiovascular disease, diabetes), medication use, and anthropometric measurements (body height, weight, waist circumference). Blood pressure was measured each minute, for a total of 8 min, in the supine position in an automatic fashion (Dinamap XL Model 9300 series device, Johnson & Johnson Medical, Tampa, FL, USA). The average of the last two measurements was taken as the ultimate blood pressure. Alcohol usage was answered with the options “no”, “1–4 per month”, “2–7 per week” 1–3 per day”, or “>4 per day”. Smoking was distinguished between “never”, “former” and “current”. Waist circumference was measured on the bare skin at the natural indentation between the 10th rib and the iliac crest.

Fasting venous blood samples were obtained, of which aliquots were stored at −80 °C and urine samples were stored at −20 °C until further analysis. Serum creatinine was measured enzymatically (Roche Modular, Roche Diagnostics, Mannheim, Germany). Serum cystatin C was measured using the Gentian Cystatin C Immunoassay (Gentian AS, Moss, Norway). Cystatin C was calibrated with known standards, according to the manufacturer’s instructions and following the guidelines of the International Federation of Clinical Chemistry Working Group for Standardization of Serum Cystatin C [25]. Triglycerides were measured enzymatically. Low-density lipoprotein (LDL) cholesterol was quantified by the Friedewald formula. Serum total cholesterol and glucose were measured with dry chemistry (Eastman Kodak, Rochester, NY, USA). Total protein levels were determined with spectrophotometry (Roche Modular, Roche Diagnostics, Roche, Mannheim, Germany). High-sensitive C-reactive protein (hs-CRP) levels were measured by nephelometry (Dade Behring Diagnostics, Marburg, Germany). In addition, 24 h urine samples were provided by participants for two days consecutively, after they were instructed both orally and in written fashion. UAE was measured in these samples, and the average was incorporated in the analysis.

### 2.3. Measurements of Oxidative Stress Biomarkers: Free Thiols, Homocysteine, Bilirubin, Gamma-Glutamyl Transferase, and HDL Cholesterol

For serum free thiols, samples were stored at −80 °C until analysis to avoid any significant changes in stability. Serum free thiol concentrations were measured after applying minor modifications [26,27]. After thawing, serum samples were diluted 4-fold, using a concentration of 0.1 mol/L Tris buffer (pH 8.2). Freezing and thawing does not cause any auto-oxidation processes that could jeopardize our measurements. Using the Varioskan microplate reader (Thermo Scientific, Breda, The Netherlands), background absorption was measured at 412 nm, together with a reference measurement at 630 nm. Following this, 20 μL of 1.9 mmol/L 5,5′-dithio-bis(2-nitrobenzoic acid) (DTNB, Ellman’s Reagent, CAS-number 69-78-3, Sigma Aldrich Corporation, St. Louis, MO, USA) in 0.1 M phosphate buffer (pH 7.0) was added to the samples, and the absorbance was measured again after the samples were incubated for 20 min at room temperature. Final concentrations of serum free thiols were established by parallel measurements of an L-cysteine (CAS-number 52-90-4, Fluka Biochemika, Buchs, Switzerland) calibration curve (concentration range from 15.625 to 1000 μmol/L) in 0.1 M Tris/10 mM EDTA (pH 8.2). Intra- and interday coefficients of variation (CV) of all measurement values were below 10%. Lastly, serum free thiol concentrations were adjusted to total serum protein levels (measured according to standard procedures), by calculating the free thiol/total protein ratio (μmol/g of protein). This adjustment was performed as serum proteins harbor the largest number of free thiols, and, therefore, largely determine the levels of potentially detectable free thiols. Homocysteine concentrations were measured on a Roche Cobas analyzer (Roche Diagnostics). HDL cholesterol levels were determined by using a homogeneous method (direct HDL; Aeroset System; Abbott Laboratories, Abbott Park, IL, USA) [14]. Plasma total bilirubin was measured by a colorimetric assay (2,4-dichloroaniline reaction; Merck MEGA, Darmstadt, Germany), with the detection limit being 1.0 mmol/L [16]. Serum γ-GT levels were measured by an enzymatic colorimetric method (Roche Modular *p*; Roche Diagnostics, Mannheim, Germany) [19].

### 2.4. Study Outcomes and Definitions

The primary study outcomes were the occurrence of cardiovascular (CV) events and all-cause mortality. Both fatal and non-fatal CV events were considered, and comprised a group containing cases of acute myocardial infarction, acute or subacute ischemic heart disease, coronary artery bypass grafting, percutaneous transluminal coronary angioplasty, intracerebral hemorrhage, other intracranial hemorrhages, subarachnoid hemorrhage, stenosis and occlusion of precerebral or cerebral arteries, and other vascular interventions, such as carotid endarterectomy, aorta peripheral bypass surgery, or percutaneous transluminal femoral angioplasty. Outcome data were retrieved from the Dutch National Registry of all hospital discharge diagnoses (Prismant), and this information was classified according to the International Statistical Classification of Diseases (ICD-10) and the International Classification of Health Interventions [28]. The estimated glomerular filtration rate (eGFR) was calculated by using the combined creatinine cystatin C-based Chronic Kidney Disease Epidemiology Collaboration (CKD-EPI) equation [29]. Type 2 diabetes was defined as a fasting glucose concentration ≥ 7.0 mmol/L or the use of oral antidiabetics, following the American Diabetes Association (ADA) guidelines. Hypertension was defined as systolic blood pressure (SBP) of ≥140 mmHg, a diastolic blood pressure (DBP) of ≥90 mHg, or both, as well as the use of antihypertensive agents. Hypercholesterolemia was defined by serum total cholesterol levels of ≥6.5 mmol/L, serum HDL cholesterol levels of ≤0.9 mmol/L, or the use of lipid-lowering drugs.

### 2.5. Statistical Analysis

Baseline demographic, clinical, and laboratory data of the study population are presented as mean ± standard deviation (SD), median (interquartile range, IQR), or as proportions *n*, with corresponding percentages (%). The assessment of normality was performed by a visual check of normal probability (Q-Q) plots and histograms. Differences between men and women for continuously distributed variables were tested using independent sample *t*-tests or Mann–Whitney *U*-tests, while for categorical variables, Chi-squared tests were performed, as appropriate. To identify the factors that were independently associated with serum free thiol levels, univariable and multivariable linear regression analyses were performed. Standardized beta (β) coefficients and corresponding *p*-values, derived from the linear regression analysis, were reported, in order to indicate the strength, direction, and statistical significance of the associations. Standardized β-coefficients represent the difference in biomarker levels per 1 SD increment for continuous variables, and the difference in biomarker levels in comparison to the specific reference group, in the case of categorical variables. Assumptions of residual normality and of homoscedasticity for linear regression were fulfilled. Biomarker levels were ^2^log-transformed prior to further analysis, in order to facilitate the results’ interpretation (expressed as per doubling). Survival distributions were created for tertiles of serum free thiol, homocysteine, gamma-GT, HDL cholesterol, and bilirubin levels by using Kaplan–Meier survival analysis. Survival time was calculated from baseline (at time of serum sampling) to the last visit, the occurrence of a cardiovascular (CV) event, death, or 1 January 2011 (end of follow-up). Cox proportional hazards regression analyses were used to assess the prospective sex-specific associations between the studied biomarkers and the risk of CV events, as well as all-cause mortality. Results from Cox proportional hazards regression models are expressed as hazard ratios (HRs). The proportionality of hazard assumption was fulfilled for all predictor variables. Multivariable Cox proportional hazards regression models were built, in order to adjust for potential confounding variables. The discriminative capacities of the Cox proportional hazards regression models were evaluated with Harrell C-statistics. Likelihood ratio (LHR) tests were used to investigate the potential incremental predictive value of selected biomarkers, in terms of clinical risk factors, with tests conducted separately for men and women. Data analysis was performed using SPSS Statistics 28.0 (SPSS Inc., Chicago, IL, USA), and data visualization was performed using RStudio (v.4.0.2) and Python (v.3.9.0, Python Software Foundation) using the *pandas* (v.1.2.3), *numpy* (v.1.20.0), *matplotlib* (v.3.4.1), and *seaborn* (v.0.11.1) packages. Two-tailed *p*-values of ≤0.05 were considered statistically significant.

### 2.6. Selection of Potentially Confounding Factors: The Directed Acyclic Graph (DAG)

**Figure 1:**
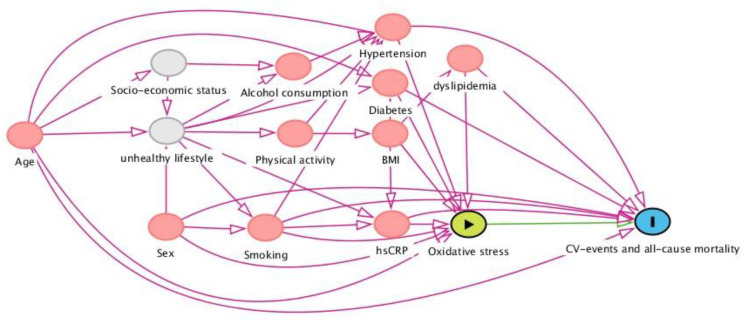
Directed acyclic graph (DAG), demonstrating the hypothesized causal relationships that underlie the investigated associations between oxidative stress biomarkers and cardiovascular events and all-cause mortality in the general population. Based on this framework, a distinct set of confounding variables was selected and subsequently conditioned for in the statistical analysis (see text). Abbreviations: BMI, body mass index; hs-CRP, high-sensitive C-reactive protein.

In order to determine potentially confounding variables that need conditioning in the prospective analyses of associations between biomarkers and study outcomes (CV events and all-cause mortality), a directed acyclic graph (DAG) was constructed (Figure 1). DAGs are causal models that serve as a theoretical basis for pre-defining the involved causal mechanisms that are hypothesized to underlie the variables at hand. The DAG depicts arrows that represent the hypothesized causal (direct) effects between variables, whereas the absence of such arrows represents the assumption of no such direct effect. In this study, we aimed to estimate the association between oxidative stress biomarkers and the risk of CV events and all-cause mortality, for which a distinct set of potentially confounding variables was identified and conditioned, in order to achieve an unconfounded effect estimate in the statistical analysis. Based on this DAG, the following variables warranted conditioning in the analysis: age, sex, smoking, total cholesterol, history of diabetes, and systolic blood pressure.

## 3. Results

### 3.1. Baseline Cohort Characteristics

Baseline population characteristics and laboratory parameters are described in Table 1, for the total population, as well as specifically for men and women. A total of 5955 research participants (2917 men and 3038 women) were included in the analyses. Baseline levels of protein-adjusted FT, homocysteine, γ-GT. and bilirubin were higher in men than in women (*p* < 0.001), whereas HDL cholesterol levels were higher in women than in men (*p* < 0.001).

### 3.2. Cross-Sectional Associations between Biomarkers and Baseline Characteristics

Univariable and multivariable linear regression analyses were performed, in order to study the cross-sectional associations between oxidative stress biomarkers and relevant study population characteristics (Figure S1, Tables S1–S5). In multivariable analyses, age was negatively associated with protein-adjusted FT (St. β = −0.126, *p* < 0.001). BMI was negatively associated with homocysteine (St. β = −0.138, *p* < 0.001) and gamma-GT (St. β = −0.055, *p* = 0.036). Waist circumference showed a strong inverse association with HD cholesterol levels (St. β = −0.302, *p* < 0.001), while being positively associated with gamma-GT and homocysteine levels (St. β = 0.127, *p* < 0.001, and St. β = 0.145, *p* < 0.001, respectively). Renal function (eGFR) was positively correlated with protein-adjusted FT (St. β = 0.192, *p* < 0.001), but was inversely correlated with homocysteine (St. β = −0.234, *p* < 0.001). Triglycerides were positively associated with protein-adjusted FT and gamma-GT (St. β = 0.107, *p* < 0.001 and St. β = 0.156, *p* < 0.001), while being inversely associated with bilirubin and HDL cholesterol (St. β = −0.077, *p* < 0.001 and St. β = −0.315, *p* < 0.001). Remaining associations are presented in Tables S1–S5 and Figure S1.

### 3.3. Sex-Specific Prospective Associations between Oxidative Stress Biomarkers and CV Events and All-Cause Mortality

**Figure 2:**
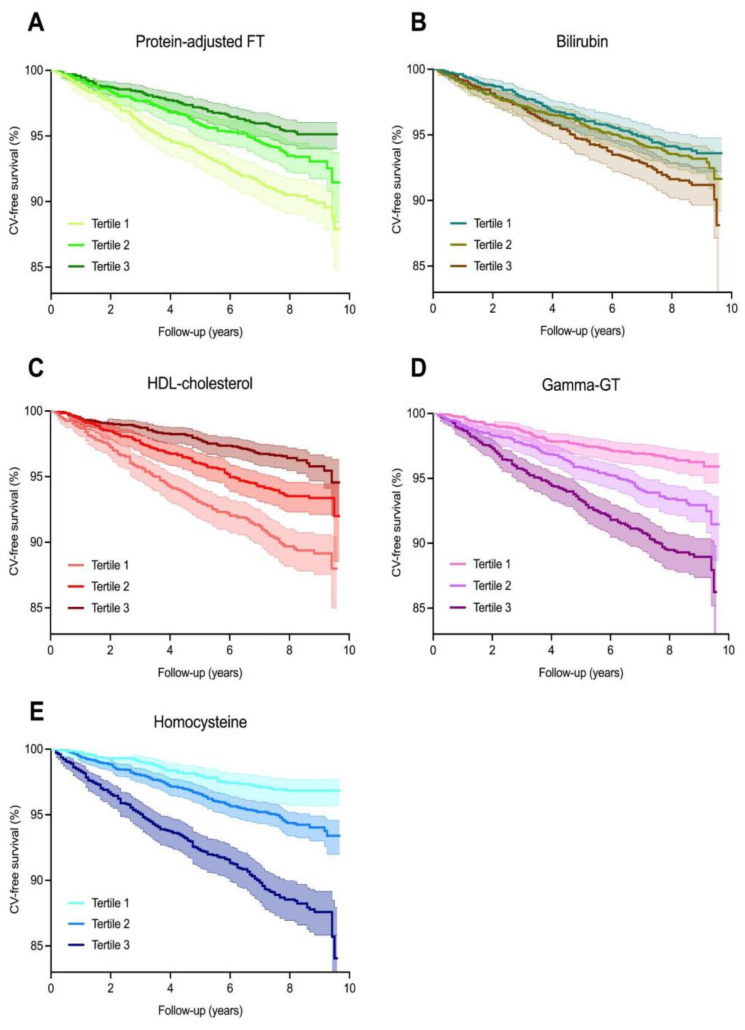
Kaplan–Meier survival distributions for tertiles of oxidative stress biomarkers. (**A**–**E**) Kaplan–Meier survival curves representing cardiovascular disease-free survival, with the highest rates of cardiovascular events occurring in the lowest tertiles of protein-adjusted FT (log-rank test, *p* < 0.001) and HDL cholesterol (*p* < 0.001) levels, and the highest tertiles of gamma-GT (*p* < 0.001) and homocysteine (*p* < 0.001) levels.

**Figure 3:**
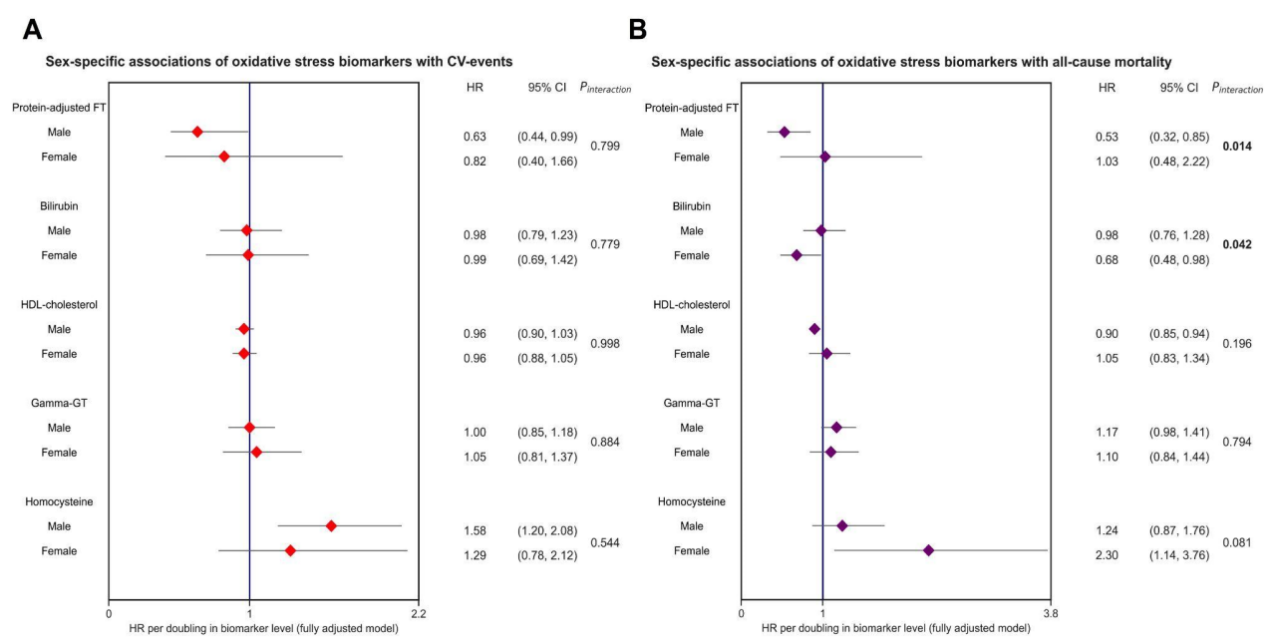
Forest plot showing sex-specific associations of oxidative stress biomarkers and the risk of CV events and all-cause mortality in the general population. Both panels demonstrate hazard ratios (HRs, red and purple dots), with corresponding 95% confidence intervals (CIs) (lines). The strongest and most statistically significant associations were observed for protein-adjusted FT and homocysteine levels, in relation to both CV events and all-cause mortality. All models were adjusted for confounding variables based on the DAG framework (see Figure 1, corresponding with *Model 4*), including age, sex, current smoking, systolic blood pressure, total cholesterol, and history of diabetes. Bold *p*-values indicate statistically significant sex interactions. Abbreviations: CI, confidence interval; FT, free thiols; GT, gamma-glutamyl transferase; HDL, high-density lipoprotein; HR, hazard ratio.

Over an average follow-up of 7.7 (±2.0) years, 402 (6.8%) CV events occurred. The highest rate of CV events was observed in participants who were within the lowest tertiles of protein-adjusted FT and HDL cholesterol levels, the second tertile of bilirubin levels, and the highest tertiles of gamma-GT and homocysteine levels. Kaplan–Meier survival analysis revealed significant differential survival distributions between tertiles of protein-adjusted FT (log-rank test, *p* < 0.001), bilirubin (*p* < 0.05), HDL cholesterol, gamma-GT, and homocysteine (all *p* < 0.001) (Figure 2). Cox proportional hazards regression analyses revealed that protein-adjusted FT, γ-GT, and homocysteine were significantly associated with the risk of CV events (all *p* < 0.001, *Model 1*, Table 2) in both sexes, while HDL cholesterol was also significantly associated with the risk of CV events in women (*Model 1*, *p* = 0.014). When adjusting for age, history of diabetes, systolic blood pressure, BMI, total cholesterol, current smoking, and hs-CRP levels (DAG-based confounding factors), protein-adjusted FT and homocysteine levels remained significantly associated with the risk of CV events in men (*Model 4*, hazard ratio (HR) 0.63 [95% CI: 0.40–0.99], *p* = 0.045 and HR 1.58 [1.20–2.08], *p* = 0.001, respectively), whereas none of the biomarkers were significantly associated with the risk of CV events in women after full adjustment for confounding factors (Figure 3).

During follow-up, a total of 316 (5.3%) participants died. Protein-adjusted FT, HDL cholesterol, γ-GT, and homocysteine levels were significantly associated with the risk of all-cause mortality in both sexes (Table 2, *Model 1*, all *p* < 0.05). However, when these associations were adjusted for the selected confounding factors, only protein-adjusted FT and HDL cholesterol remained significantly associated with the risk of all-cause mortality in men (*Model 4*, HR 0.52 [0.32–0.85], *p* = 0.009 and HR 0.90 [0.85–0.94], *p* < 0.001, respectively), while bilirubin and homocysteine were significantly associated with all-cause mortality in women (*Model 4*, HR 0.68 [0.48–0.98], *p* = 0.040 and HR 2.30 [1.14–3.76], *p* < 0.001, respectively). Interaction analyses revealed significant effect modifications for the associations between protein-adjusted FT, as well as bilirubin, and the risk of all-cause mortality by sex (both *p* < 0.05), with the strongest associations in males being for protein-adjusted FT levels, and those in females being for bilirubin (Figure 3).

### 3.4. Incremental Value of Oxidative Stress Biomarkers over Clinical Risk Factors

Subsequently, we aimed to evaluate the added value of the biomarkers over a base risk model containing the selected confounding factors (derived from the DAG) in predicting the risk of CV events (Table 3). In general, the addition of the biomarkers did not substantially improve model discrimination across both sexes. In men, the greatest increment was observed after adding homocysteine (ΔC-statistic: 0.005, *p* < 0.01), followed by protein-adjusted FT (ΔC-statistic: 0.002, *p* < 0.05). The addition of both homocysteine and protein-adjusted serum free thiols did not yield an improved model discrimination when compared to either one of these biomarkers (LHR Chi-square: 2.35, *p* = 0.125). For bilirubin, HDL cholesterol, and γ-GT, no significant model discrimination or model fit was observed. In women, none of the biomarkers significantly improved model discrimination or model fit.

## 4. Discussion

The current study indicates significant associations between protein-adjusted FT, bilirubin, HDL cholesterol, γ-GT, and homocysteine as oxidative stress biomarkers, as well as highlights the risk of CV events and all-cause mortality in individuals from the general population. Baseline levels of protein-adjusted FT, homocysteine, γ-GT, and bilirubin were higher in men than in women (*p* < 0.001), whereas HDL cholesterol levels were higher in women (*p* < 0.001). After adjustment for potentially confounding factors, protein-adjusted FT and homocysteine levels were significantly associated with the risk of cardiovascular (CV) events, though only in men. After adjustment for selected confounding factors, only protein-adjusted FT and HDL cholesterol levels remained significantly associated with the risk of all-cause mortality in men, while bilirubin and homocysteine remained significantly associated with all-cause mortality in women. These results confirm the strength of protein-adjusted FT as being a reliable biomarker for oxidative stress, in the context of cardiovascular disease and all-cause mortality, while also highlighting the importance of other oxidative stress-related biomarkers, such as homocysteine, HDL cholesterol, and bilirubin. Combining both the protein-adjusted free thiol and homocysteine levels in a statistical model, however, did not yield a significantly different outcome.

Previous studies have also found that serum free thiols positively associate with CV events and mortality in the general population, and that they comprise a useful biomarker for oxidative stress in the general population [30]. Additionally, total thiol levels have also been shown to strongly associate with CV events and all-cause mortality [31]. However, in our study, we found this association to only remain significant in men for both CV events and all-cause mortality, after adjusting for potentially confounding factors. Previously we have investigated the associations between this biomarker and CV events in the female population, where we also concluded that these associations lost their significance after correcting for age, suggesting that age-related factors play an important role in the associations between oxidative stress and the occurrence of cardiovascular disease, especially in women [20]. This sex-related difference could potentially be explained by menopausal differences. Menopause is believed to be accompanied by oxidative stress, which may at least partially be driven by reduced estrogen production, which has known antioxidant effects [32]. We hypothesize that this could lead to comparatively higher levels of FT in men than in women.

Homocysteine is known to be associated with an increased risk of cardiovascular disease and mortality [31,32,33,34,35]. More recently, it has also been found to upregulate oxidative stress via enhancing GPX4 (glutathione peroxidase) methylation [12]. Another study found hyperhomocysteinemia to be an independent risk factor of coronary heart disease, but did not draw sex-specific conclusions [36]. Our findings of homocysteine being positively associated with mortality were confirmed by another study, though they did not examine sex-specific associations, whereas we found the association to only remain statistically significant in women, after correcting for potentially confounding factors [37]. Homocysteine concentrations are significantly higher in men than in women, which could relate to changes in renal function and creatinine concentrations [38]. Moreover, homocysteine is reduced by estrogen, both directly, through effects on homocysteine synthesis, and indirectly, through its effect on gene expression [39]. We theorize that this could explain why CV events were associated significantly with homocysteine levels in men only, as women might benefit from this estrogen-related reduction until menopausal age. Menopause appears to play an important role in regulating homocysteine concentrations in women, which could potentially explain why there was a significant association between homocysteine levels and mortality in women in our study [40,41].

Upon combining FT and homocysteine in a statistical model to predict CV events and mortality, we did not yield any significantly different new results. Further research into combining multiple biomarkers for the prediction of CV events and all-cause mortality could prove to be interesting if shown to increase the accuracy of prediction.

After correcting for potentially confounding factors, we found HDL cholesterol levels to significantly inversely associate with only all-cause mortality in men. Several studies draw conclusions about HDL cholesterol and all-cause mortality. Total and small HDL particle concentrations strongly and independently predicted 3 month mortality in acute heart failure patients [42]. Interestingly, there is a paradoxical association of high HDL cholesterol levels with high mortality in the general population [43]. This association is U-shaped, meaning there appears to be an increased risk of all-cause mortality at both the lowest and highest concentrations of HDL cholesterol [44]. A different study attributed the paradoxical association to genetic variations in certain mutations that were previously associated with an increased risk of coronary heart disease, as well as high concentrations of HDL cholesterol [45,46]. In a pooled analysis of 37 prospective cohort studies, researchers further supported the U-shaped association of both extremely high and low HDL cholesterol levels with an increased risk of all-cause mortality [47]. Finally, pharmacologically increasing levels of HDL cholesterol does not seem to reduce CV events in substantial trials of the three agents they investigated [48].

A previous PREVEND study also showed a modest log-linear inverse association between circulating total bilirubin levels and cardiovascular events that was independent of established risk factors [16]. However, they did not find evidence of sex differences significantly modifying the bilirubin–CV event association, which is similar to our study, where we did not find any significant associations after correcting for potentially confounding factors. We did, however, demonstrate an inverse association between bilirubin and all-cause mortality, but only in women. Other researchers also found a strong negative association between plasma bilirubin levels and both total and cancer mortality, albeit they did not investigate sex-related differences [49].

After correcting for potentially confounding factors, γ-GT seemed to associate, in both men or women, with neither CV events nor mortality in our study. However, this biomarker has been found to positively correlate with both CV events and all-cause mortality in the past [50,51,52]. This difference in conclusions may be caused by differences in study populations, as another study with data from the PREVEND study came to a similar conclusion as ours, in that γ-GT correlates significantly with CV events only until correction for potentially confounding factors [19]. Further large-scale research would be required to untangle this.

One of the core strengths of the present study is the size of the study population. The PREVEND study features information on dozens of phenotypic variables from thousands of people. Furthermore, this study was of a prospective nature, encompassing almost 10 years of follow-up, granting insight into the development of cardiovascular disease and rate of mortality during this period. Additionally, our study investigated five different biomarkers of oxidative stress within the same population, which allowed us to better compare the strength of the individual biomarkers than studies that may only look at one or two at a time. However, any potential limitations of our study should also be taken into account. The data used in this study contained a vast majority of study subjects of Caucasian ethnicity, which makes it difficult to draw any conclusions that can be applied to other ethnicities. A similar caution about the generalizability should be applied to other data points that may be related to the geographical location of the PREVEND study population. As with most databases that are partially survey-based, the self-reported nature of some variables could lead to over- or underestimation by participants. In addition, the study endpoint’s definition of CV events did not include heart failure (HF) as an outcome, since it is pathophysiologically distinct, and thus requires further study. Furthermore, the parameters regarding the biomarkers we investigated in our study were limited by previously generated biomarker data and, thus, only a few were eligible for analysis. In this regard, the potential value of γ-GT and total bilirubin levels as oxidative stress biomarkers needs to be cautiously observed in future studies, since their levels may be impacted by the presence of hepatobiliary disease. There was an insufficient volume of collected from the study participants that could otherwise have been used to extend our panel of oxidative stress markers. It is possible that because of this, we might have missed alternative redox biomarkers that could have an equal or better predictive value in relation to CV events and all-cause mortality than those we investigated. An unbiased approach, such as investigating a combination of key components of the redox metabolome, would be preferable, as read-outs of multiple redox-regulated metabolic pathways would be combined. However, such “redox metabolomics” approaches are still constrained by several (mostly methodological) issues, which would have to be further explored first [53].

Additionally, further research into possibilities for therapeutic modulation of redox biomarkers could contribute to a decrease in oxidative stress, with all the health benefits that come with that. For example, serum free thiols are amenable to nutritional or therapeutic intervention, and may be of use in the therapeutic modulation of redox status in various conditions [54,55].

## 5. Conclusions

In conclusion, we demonstrated that both protein-adjusted FT and homocysteine associate significantly with the risk of CV events and all-cause mortality. This further highlights the predictive qualities of these two biomarkers of oxidative stress for disease outcomes in the general population. Further studies are required to validate these observations and examine associations across different populations and relevant subgroups.

## Figures and Tables

**Table 1:** Population characteristics and laboratory parameters.

Variable	Total	Men	Women	*p*-Value
	*n* = 5955	*n* = 2917	*n* = 3038	
Age (years)	51.6 [43.3;61.7]	52.4 [43.8;63.9]	50.9 [43.0;59.7]	<0.001
Ethnicity Caucasian, *n* (%) Asian, *n* (%) African, *n* (%) Other, *n* (%)	5676 (95.3) 120 (2.0) 56 (0.9) 63 (1.1)	2779 (95.3) 61 (2.1) 25 (0.9) 34 (1.2)	2897 (95.4) 59 (1.9) 31 (1.0) 29 (1.0)	0.750
BMI (kg/m^2^)	26.0 [23.6;28.9]	26.4 [24.2;28.8]	25.6 [23.1;28.9]	<0.001
Waist circumference (cm)	91 [82;100]	96 [89;103]	85 [78;94]	<0.001
Smoking Never, *n* (%) Former, *n* (%) Current, *n* (%)	1757 (29.5) 2472 (41.5) 1655 (27.8)	756 (25.9) 1322 (45.3) 802 (27.5)	1001 (32.9) 1150 (37.9) 853 (28.1)	0.640
Alcohol use No, *n* (%) Yes, *n* (%)	1454 (24.4) 4450 (74.7)	502 (17.2) 2386 (81.8)	952 (31.3) 2064 (67.9)	<0.001
Blood pressure SBP (mmHg) DBP (mmHg)	123 [112;136] 73 [67;79]	127 [117;139] 76 [70;82]	117 [108;131] 69 [64;76]	<0.001 <0.001
**Comorbidity**				
CVD history, *n* (%) Hypertension, *n* (%) Non-insulin-treated type 2 diabetes, *n* (%)	217 (3.6) 1717 (28.8) 144 (2.4)	155 (5.3) 932 (32.0) 74 (2.5)	62 (2.0) 785 (25.8) 70 (2.3)	<0.001 <0.001 0.560
**Study biomarkers**				
Serum free thiols (μmol/g)	5.05 ± 1.02	5.12 ± 1.03	4.98 ± 1.01	<0.001
Bilirubin (umol/L)	7.0 [5.0;9.0]	8.0 [6.0;10.0]	6.0 [5.0;8.0]	<0.001
Homocysteine (umol/L)	12.0 [10.0;14.0]	13.0 [11.0;15.0]	11.0 [9.0;13.0]	<0.001
HDL cholesterol (mg/dL)	47.4 [40.2;56.0]	42.5 [36.9;49.3]	52.5 [45.3;60.6]	<0.001
Gamma-GT (U/L)	23.0 [16.0;37.0]	30.0 [21.0;47.0]	18.0 [13.0;27.0]	<0.001
**Laboratory parameters**				
Hemoglobin (mmol/L)	8.5 ± 0.8	9.0 ± 0.6	8.1 ± 0.6	<0.001
hs-CRP (mg/L)	1.3 [0.6;2.9]	1.2 [0.6;2.7]	1.4 [0.6;3.2]	0.002
Albumin (g/L)	44.0 [42.0;45.0]	44.0 [43.0;46.0]	43.0 [42.0;45.0]	<0.001
Creatinine (μmol/L)	82.1 [73.9;92.4]	90.4 [83.2;98.9]	74.9 [68.8;82.1]	<0.001
eGFR (mL/min/1.73 m^2^)	94.5 [82.2;104.7]	94.6 [82.6;105.4]	94.5 [82.0;104.1]	0.176
Total cholesterol (mmol/L)	5.4 [4.7;6.1]	5.4 [4.8;6.1]	5.4 [4.7;6.1]	0.904
LDL cholesterol (mmol/L)	3.4 [2.7;4.2]	3.5 [2.7;4.2]	3.3 [2.7;4.1]	0.407
Triglycerides (mg/dL)	97.2 [70.5;140.8]	109.4 [77.1;157.9]	89.4 [65.7;123.1]	<0.001
Glucose (mmol/L)	4.7 [4.4;5.2]	4.8 [4.5;5.3]	4.7 [4.3;5.1]	<0.001
**Follow-up (10 years)**				
CV events, *n* (%)	402 (6.8)	289 (9.9)	113 (3.7)	<0.001
Mortality, *n* (%)	316 (5.3)	220 (7.5)	96 (3.2)	<0.001

**Table 2:** Cox proportional hazards regression analyses for associations between oxidative stress biomarkers and the risk of incident CVD and all-cause mortality.

		FT	Bilirubin	HDL	γ-GT	HCys
**Cardiovascular Events (CV)**						
Model 1 *	M	0.33 [0.24–0.47], ***p* < 0.001**	0.97 [0.81–1.16], *p* = 0.758	0.95 [0.89–1.00], *p* = 0.084	1.25 [1.11–1.40], ***p* < 0.001**	2.04 [1.70–2.44], ***p* < 0.001**
	F	0.36 [0.22–0.60], ***p* < 0.001**	0.94 [0.71–1.24], *p* = 0.657	0.91 [0.85–0.98], ***p* = 0.014**	1.44 [1.20–1.73], ***p* < 0.001**	2.31 [1.61–3.32], ***p* < 0.001**
Model 2	M	0.68 [0.47–0.98], ***p* = 0.039**	0.86 [0.71–1.03], *p* = 0.104	0.93 [0.88–0.99], ***p* = 0.026**	1.08 [1.06–1.09], ***p* < 0.001**	1.65 [1.32–2.06], ***p* < 0.001**
	F	0.80 [0.43–1.46], *p* = 0.458	0.81 [0.60–1.10], *p* = 0.182	0.95 [0.88–1.02], *p* = 0.142	1.22 [1.00–1.50], *p* = 0.056	1.50 [0.99–2.27], *p* = 0.053
Model 3	M	0.76 [0.52–1.11], *p* = 0.152	0.957 [0.78–1.17], *p* = 0.665	0.96 [0.90–1.02], *p* = 0.203	1.11 [0.97–1.27], *p* = 0.142	1.62 [1.29–2.05], ***p* < 0.001**
	F	0.82 [0.45–1.49], *p* = 0.514	0.91 [0.67–1.24], *p* = 0.552	0.96 [0.89–1.04], *p* = 0.368	1.07 [0.86–1.34], *p* = 0.555	1.27 [0.83–1.94], *p* = 0.264
Model 4	M	0.63 [0.40–0.99], ***p* = 0.045**	0.98 [0.79–1.23], *p* = 0.879	0.96 [0.90–1.03], *p* = 0.301	1.00 [0.85–1.18], *p* = 0.982	1.58 [1.20–2.08], ***p* = 0.001**
	F	0.82 [0.40–1.66], *p* = 0.574	0.99 [0.69–1.42], *p* = 0.940	0.96 [0.88–1.05], *p* = 0.386	1.05 [0.81–1.37], *p* = 0.699	1.29 [0.78–2.12], *p* = 0.320
**All-cause mortality**						
Model 1	M	0.22 [0.15–0.32], ***p* < 0.001**	1.11 [0.90–1.36], *p* = 0.335	0.91 [0.86–0.96], ***p* < 0.001**	1.19 [1.04–1.37], ***p* = 0.014**	2.02 [1.64–2.49], ***p* < 0.001**
	F	0.41 [0.23–0.72], ***p* = 0.002**	0.82 [0.61–1.09], *p* = 0.173	0.92 [0.85–1.00], ***p* = 0.050**	1.34 [1.09–1.65], ***p* = 0.006**	3.21 [2.22–4.65], ***p* < 0.001**
Model 2	M	0.60 [0.40–0.90], ***p* = 0.015**	0.86 [0.69–1.08], *p* = 0.864	0.89 [0.84–0.93], ***p* < 0.001**	1.32 [1.14–1.52], ***p* < 0.001**	1.361 [0.98–1.74], *p* = 0.065
	F	1.06 [0.54–2.08], *p* = 0.876	0.67 [0.49–0.93], ***p* = 0.016**	0.95 [0.88–1.04], *p* = 0.272	1.11 [0.88–1.40], *p* = 0.365	2.23 [1.46–3.39], ***p* < 0.001**
Model 3	M	0.66 [0.43–1.01], *p* = 0.056	0.94 [0.74–1.19], *p* = 0.606	0.89 [0.85–0.94], ***p* < 0.001**	1.29 [1.10–1.50], ***p* = 0.001**	1.21 [0.89–1.63], *p* = 0.221
	F	0.97 [0.50–1.89], *p* = 0.926	0.73 [0.52–1.01], *p* = 0.059	0.96 [0.88–1.04], *p* = 0.329	1.03 [0.81–1.32], *p* = 0.793	1.95 [1.27–2.98], ***p* = 0.002**
Model 4	M	0.52 [0.32–0.85], ***p* = 0.009**	0.98 [0.76–1.28], *p* = 0.903	0.90 [0.85–0.94], ***p* < 0.001**	1.17 [0.98–1.41], *p* = 0.089	1.24 [0.87–1.76], *p* = 0.241
	F	1.03 [0.48–2.22], *p* = 0.940	0.68 [0.48–0.98], ***p* = 0.040**	1.05 [0.83–1.34], *p* = 0.684	1.10 [0.84–1.44], *p* = 0.475	2.30 [1.14–3.76], ***p* < 0.001**

* Model 1, crude model. Model 2, Model 1 with adjustment for age. Model 3, Model 2 with adjustment for history of diabetes, systolic blood pressure, BMI, total cholesterol, and current smoking. Model 4, Model 3 with adjustment for hs-CRP. **Bold** *p*-values indicate statistical significance. Abbreviations: HCys, homocysteine; HDL, high-density lipoprotein; HR, hazard ratio; FT, free thiols; γ-GT, gamma-glutamyl transferase.

**Table 3:** Sex-specific incremental value of oxidative stress biomarkers over clinical risk factors in predicting the risk of CV events and all-cause mortality.

Biomarker	Men	*p*-Value	Women	*p*-Value
**Cardiovascular (CV) Events**				
Base model *, C-statistic	0.772	-	0.806	-
−2LL	3082.4	-	1215.3	-
Base model + FT, C-statistic	0.774	-	0.805	-
ΔC-statistic	0.002	-	−0.001	-
−2LL	3078.5	-	1214.9	-
LHR Chi-square	3.90	0.048	0.31	0.577
Base model + bilirubin, C-statistic	0.772		0.806	
ΔC-statistic	<0.001		<0.001	
−2LL	3082.4		1215.2	
LHR Chi-square	0.02	0.879	0.006	0.940
Base model + HDL, C-statistic	0.772		0.807	
ΔC-statistic	<0.001		0.001	
−2LL	3081.6		1214.7	
LHR Chi-square	0.84	0.358	0.60	0.439
Base model + γ-GT, C-statistic	0.772		0.806	
ΔC-statistic	<0.001		<0.001	
−2LL	3082.4		1215.1	
LHR Chi-square	0.001	0.982	0.15	0.700
Base model + HCys, C-statistic	0.777		0.804	
ΔC-statistic	0.005		−0.002	
−2LL	3072.6		1214.3	
LHR Chi-square	9.79	0.002	0.97	0.325
Base model + HCys + FT, C-statistic	0.779		0.803	
ΔC-statistic	0.002		−0.003	
−2LL	3070.3		1214.1	
LHR Chi-square	12.1	0.002	1.16	0.560

* Base model includes adjustments for age, history of diabetes, systolic blood pressure, BMI, total cholesterol levels, current smoking, and hs-CRP. Abbreviations: −2LL, −2 log-likelihood; FT, free thiols; γ-GT, gamma-glutamyl transferase; HCys, homocysteine; HDL, high-density lipoprotein; LHR, likelihood ratio test.

## Data Availability

The datasets generated for this study are available on request to the corresponding author.

## Supplementary Materials

The following supporting information can be downloaded at: https://www.mdpi.com/article/10.3390/antiox12030690/s1, Figure S1: Heatmap depicting crude associations between oxidative stress biomarkers and baseline demographic, anthropometric, clinical, and laboratory characteristics; Table S1: Linear regression of protein-adjusted free thiols; Table S2: Linear regression of bilirubin; Table S3: Linear regression of homocysteine; Table S4: Linear regression of gamma-GT; Table S5: Linear regression of high-density lipoprotein.
