# Supplementary material for: A Sex-Specific Comparative Analysis of Oxidative Stress Biomarkers Predicting the Risk of Cardiovascular Events and All-Cause Mortality in the General Population: A Prospective Cohort Study
Source: Antioxidants (Basel). 2023 Mar 10;12(3):690. doi: 10.3390/antiox12030690 (PMC10044882; doi:10.3390/antiox12030690)
Supplement: Supplementary file 1 [file antioxidants-12-00690-s001.zip › antioxidants-2235055-supplementary.pdf]

Figure S1

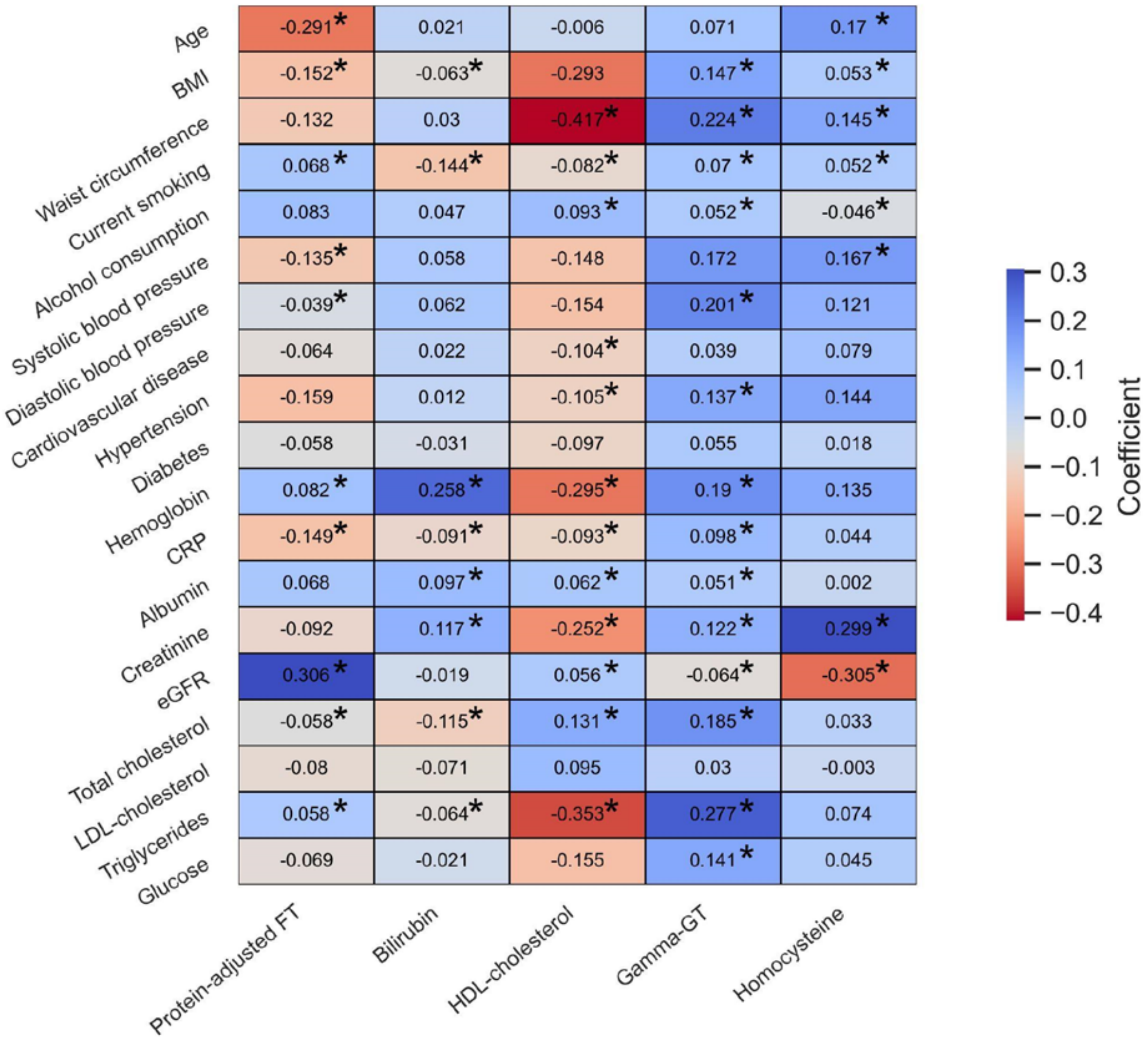

Figure S1. Heatmap depicting crude associations between oxidative stress biomarkers and baseline demographic, anthropometric, clinical, and laboratory characteristics. Numbers represent standardized beta-coefficients derived from univariable linear regression analysis, colored by the direction of the association (blue: positive; red: negative). Asterisks (*) indicate statistically significant associations in multivariable analyses (see Supplementary Tables S1-S5).

Table S1: Linear regression of protein-adjusted free thiols

Variable	Univariable analysis		Multivariable analysis	
	St. Beta	*P*-value	St. Beta	*P*-value
Age (years)	-0.291	**<0.001**	-0.126	**<0.001**
BMI (kg/m^2)	-0.152	**<0.001**	-0.091	**<0.001**
Waist circumference (cm)	-0.132	**<0.001**		
Current smoking (%)	0.068	**<0.001**	0.038	**0.008**
Alcohol use (%)	0.083	**<0.001**		
Systolic blood pressure (mmHg)	-0.135	**<0.001**	-0.078	**<0.001**
Diastolic blood pressure (mmHg)	-0.039	**0.003**	0.072	**<0.001**
CVD history (%)	-0.064	**<0.001**		
Hypertension (%)	-0.159	**<0.001**		
Diabetes (%)	-0.058	**<0.001**		
Laboratory parameters				
Hemoglobin (mmol/L)	0.082	**<0.001**	0.089	**<0.001**
hs-CRP (mg/L)	-0.149	**<0.001**	-0.082	**<0.001**
Albumin (g/L)	0.068	**<0.001**		
Creatinine (µmol/L)	-0.092	**<0.001**		
eGFR (mL/min/1.73m^2)	0.306	**<0.001**	0.192	**<0.001**

Total cholesterol (mmol/L)	-0.058	**<0.001**	-0.037	**0.015**
LDL-cholesterol (mmol/L)	-0.080	0.053		
Triglycerides (mg/dL)	0.058	**<0.001**	0.107	**<0.001**
Glucose (mmol/L)	-0.069	**<0.001**		

Table S2: Linear regression of bilirubin

Variable	Univariable analysis		Multivariable analysis	
	St. Beta	*P*-value	St. Beta	*P*-value
Age (years)	0.021	0.105		
BMI (kg/m^2)	-0.063	**<0.001**	-0.076	**<0.001**
Waist circumference (cm)	0.030	**0.021**		
Current smoking (%)	-0.144	**<0.001**	-0.165	**<0.001**
Alcohol use (%)	0.047	**<0.001**		
Systolic blood pressure (mmHg)	0.058	**<0.001**		
Diastolic blood pressure (mmHg)	0.062	**<0.001**		
CVD history (%)	0.022	0.087		
Hypertension (%)	0.012	0.372		
Diabetes (%)	-0.031	**0.017**		
Laboratory parameters				

Hemoglobin (mmol/L)	0.258	**<0.001**	0.300	**<0.001**
hs-CRP (mg/L)	-0.091	**<0.001**	-0.047	**<0.001**
Albumin (g/L)	0.097	**<0.001**	0.039	**0.005**
Creatinine (µmol/L)	0.117	**<0.001**	0.038	**0.009**
eGFR (mL/min/1.73m^2)	-0.019	0.152		
Total cholesterol (mmol/L)	-0.115	**<0.001**	-0.116	**<0.001**
LDL-cholesterol (mmol/L)	-0.071	0.086		
Triglycerides (mg/dL)	-0.064	**<0.001**	-0.077	**<0.001**
Glucose (mmol/L)	-0.021	0.111		

Table S3: Linear regression of homocysteine

**Variable**	**Univariable analysis**		**Multivariable analysis**	
	St. Beta	*P*-value	St. Beta	*P*-value
Age (years)	0.170	**<0.001**	-0.071	**<0.001**
BMI (kg/m^2)	0.053	**<0.001**	-0.138	**<0.001**
Waist circumference (cm)	0.145	**<0.001**	0.145	**<0.001**
Current smoking (%)	0.052	**<0.001**	0.073	**<0.001**
Alcohol use (%)	-0.046	**<0.001**	-0.039	**0.003**
Systolic blood pressure (mmHg)	0.167	**<0.001**	0.087	**<0.001**

Diastolic blood pressure (mmHg)	0.121	**<0.001**		
CVD history (%)	0.079	**<0.001**		
Hypertension (%)	0.144	**<0.001**		
Diabetes (%)	0.018	0.160		
Laboratory parameters				
Hemoglobin (mmol/L)	0.135	**<0.001**		
hs-CRP (mg/L)	0.044	**0.003**		
Albumin (g/L)	0.002	0.893		
Creatinine (µmol/L)	0.299	**<0.001**	0.145	**<0.001**
eGFR (mL/min/1.73m^2)	-0.305	**<0.001**	-0.234	**<0.001**
Total cholesterol (mmol/L)	0.033	**0.012**		
LDL-cholesterol (mmol/L)	-0.003	0.940		
Triglycerides (mg/dL)	0.074	**<0.001**		
Glucose (mmol/L)	0.045	**0.001**		

Table S4: Linear regression of gamma-GT

**Variable**	**Univariable analysis**		**Multivariable analysis**	
	St. Beta	*P*-value	St. Beta	*P*-value
Age (years)	0.071	**<0.001**		

BMI (kg/m^2)	0.147	**<0.001**	-0.055	**0.036**
Waist circumference (cm)	0.224	**<0.001**	0.127	**<0.001**
Current smoking (%)	0.070	**<0.001**	0.047	**0.002**
Alcohol use (%)	0.052	**<0.001**	0.044	**0.003**
Systolic blood pressure (mmHg)	0.172	**<0.001**		
Diastolic blood pressure (mmHg)	0.201	**<0.001**	0.058	**0.001**
CVD history (%)	0.039	**0.003**		
Hypertension (%)	0.137	**<0.001**	0.037	**0.040**
Diabetes (%)	0.055	**<0.001**		
Laboratory parameters				
Hemoglobin (mmol/L)	0.190	**<0.001**	0.050	**0.005**
hs-CRP (mg/L)	0.098	**<0.001**	0.072	**<0.001**
Albumin (g/L)	0.051	**<0.001**	0.032	**0.028**
Creatinine (μmol/L)	0.122	**<0.001**	0.048	**0.012**
eGFR (mL/min/1.73m^2)	-0.064	**<0.001**	0.067	**<0.001**
Total cholesterol (mmol/L)	0.185	**<0.001**	0.094	**<0.001**
LDL-cholesterol (mmol/L)	0.030	0.472		
Triglycerides (mg/dL)	0.277	**<0.001**	0.156	**<0.001**

Glucose (mmol/L)	0.141	**<0.001**	0.048	**0.002**

Table S5: Linear regression of high-density lipoprotein

Variable	Univariable analysis		Multivariable analysis	
	St. Beta	*P*-value	St. Beta	*P*-value
Age (years)	-0.006	0.640		
BMI (kg/m^2)	-0.293	**<0.001**		
Waist circumference (cm)	-0.417	**<0.001**	-0.302	**<0.001**
Current smoking (%)	-0.082	**<0.001**	-0.079	**<0.001**
Alcohol use (%)	0.093	**<0.001**	0.112	**<0.001**
Systolic blood pressure (mmHg)	-0.148	**<0.001**		
Diastolic blood pressure (mmHg)	-0.154	**<0.001**		
CVD history (%)	-0.104	**<0.001**	-0.038	**0.003**
Hypertension (%)	-0.105	**<0.001**	0.048	**<0.001**
Diabetes (%)	-0.097	**<0.001**		
Laboratory parameters				
Hemoglobin (mmol/L)	-0.295	**<0.001**	-0.131	**<0.001**
hs-CRP (mg/L)	-0.093	**<0.001**	-0.036	**0.005**
Albumin (g/L)	0.062	**<0.001**	0.066	**<0.001**

Creatinine (µmol/L)	-0.252	**<0.001**	-0.171	**<0.001**
eGFR (mL/min/1.73m^2)	0.056	**<0.001**	-0.134	**<0.001**
Total cholesterol (mmol/L)	0.131	**<0.001**	0.272	**<0.001**
LDL-cholesterol (mmol/L)	0.095	**0.022**		
Triglycerides (mg/dL)	-0.353	**<0.001**	-0.315	**<0.001**
Glucose (mmol/L)	-0.155	**<0.001**		
